# First-trimester fetal size, accelerated growth in utero, and child neurodevelopment in a cohort study

**DOI:** 10.1186/s12916-024-03390-3

**Published:** 2024-04-29

**Authors:** Xinmei Chen, Hongxiu Liu, Yuanyuan Li, Wenxin Zhang, Aifen Zhou, Wei Xia, Shunqing Xu

**Affiliations:** 1 Key Laboratory of Environment and Health, Ministry of Education & Ministry of Environmental Protection, and State Key Laboratory of Environmental Health, School of Public Health, Tongji Medical College, Huazhong University of Science and Technology, Wuhan, 430030 People’s Republic of China; 2 School of Environmental Science and Engineering, Hainan University, Haikou, 570228 People’s Republic of China; 3 Women and Children Medical and Healthcare Center of Wuhan, Wuhan, 430015 People’s Republic of China

**Keywords:** Child neurodevelopment, First-trimester fetal size, Intrauterine accelerated growth, Cohort study

## Abstract

**Background:**

Early pregnancy is a critical window for neural system programming; however, the association of first-trimester fetal size with children’s neurodevelopment remains to be assessed. This study aimed to explore the association between first-trimester fetal size and children’s neurodevelopment and to examine whether intrauterine accelerated growth could compensate for the detrimental effects of first-trimester restricted growth on childhood neurodevelopment.

**Methods:**

The participants were from a birth cohort enrolled from March 2014 to March 2019 in Wuhan, China. A total of 2058 fetuses with crown to rump length (CRL) (a proxy of first-trimester fetal size) measurements in the first trimester and neurodevelopmental assessment at age 2 years were included. We measured the first-trimester CRL and defined three fetal growth patterns based on the growth rate of estimated fetal weight from mid to late pregnancy. The neurodevelopment was assessed using the Bayley Scales of Infant Development of China Revision at 2 years.

**Results:**

Each unit (a Z score) increase of first-trimester CRL was associated with increased scores in mental developmental index (MDI) (adjusted beta estimate = 1.19, (95% CI: 0.42, 1.95), *P* = 0.03) and psychomotor developmental index (PDI) (adjusted beta estimate = 1.36, (95% CI: 0.46, 2.26), *P* < 0.01) at age 2 years, respectively. No significant association was observed between fetal growth rate and PDI. For children with restricted first-trimester fetal size (the lowest tertile of first-trimester CRL), those with “intrauterine accelerated growth” pattern (higher growth rates) had significantly higher MDI (adjusted beta estimate = 6.14, (95% CI: 3.80, 8.49), *P* < 0.001) but indistinguishable PDI compared to those with “intrauterine faltering growth” pattern (lower growth rates). Main limitations of this study included potential misclassification of gestational age due to recall bias of the last menstrual period and residual confounding.

**Conclusions:**

The current study suggests that restricted first-trimester fetal size is associated with mental and psychomotor developmental delay in childhood. However, in children with restricted first-trimester fetal size, intrauterine accelerated growth was associated with improved mental development but had little effect on psychomotor development. Additional studies are needed to validate the results in diverse populations.

**Supplementary Information:**

The online version contains supplementary material available at 10.1186/s12916-024-03390-3.

## Background

Restricted growth in utero is a recognized risk factor for impaired brain development [1–3]. Fetal brain development begins in the first few weeks after conception and continues throughout pregnancy [4]. The early stages of pregnancy primarily involve the development of neural tube [5]; by mid-gestation, the number of fetal brain cells continues to increase, and sensory systems begin to develop gradually [4]; in late gestation, the structure of the fetal cerebral cortex becomes progressively more complex, with richer neuronal connections [6]. It is well-known that cell proliferation is very rapid during the first trimester of gestation [7], and the first trimester is also a pivotal stage for the development of fetal neural tubes [5], suggesting that the first trimester is a critical period for neurodevelopment. However, the current epidemiological evidence regarding an association between first-trimester fetal size and childhood neurodevelopment is limited, and prior studies on the association of second-trimester and third-trimester fetal size with neurodevelopment have also given little consideration to first-trimester fetal size [8–11].

Little is known about the influence of fetal growth patterns on childhood neurodevelopment. Fetal growth patterns, such as intrauterine accelerated growth, can improve the fetal size, which offers those fetuses with restricted first-trimester fetal size a chance of being the normal size in late pregnancy or at birth. Although the restricted first-trimester fetal size may be associated with neurodevelopmental delay, whether intrauterine accelerated growth ameliorates the neurodevelopmental delay of these children with restricted first-trimester fetal size has never been studied. Previous studies have identified the risk of neurodevelopmental delay by birth size, with smaller birth sizes being at greater risk for neurodevelopmental delay [12–14]. However, assuming that intrauterine accelerated growth cannot compensate for the neurodevelopmental delay associated with restricted first-trimester growth, their normal size at late pregnancy or birth may lead to an underestimation for their risk of neurodevelopmental delay.

Therefore, taking advantage of a prospective birth cohort with serial fetal ultrasound measurements throughout pregnancy and the neurodevelopment test at age 2 years, the overall research point of the present study was to fill the gap mentioned above by investigating the associations of first-trimester fetal size and intrauterine accelerated growth with early childhood neurodevelopment. We hypothesized that (1) first-trimester fetal size would be positively associated with childhood neurodevelopment, and restricted first-trimester fetal size would be associated with neurodevelopmental delay; (2) for children with restricted first-trimester fetal size, intrauterine accelerated growth would be associated with improved neurodevelopment; (3) the above associations would be independent of birth outcomes and postnatal growth.

## Methods

### Population and study design

This study was embedded in a prospective birth cohort that recruited pregnant women from March 2014 to March 2019 in Wuhan Medical & Healthcare Center for Women and Children, Wuhan, China. Pregnant women who resided in Wuhan city with a singleton pregnancy and planned to deliver at the study hospital were invited to participate in the cohort [15]. We initially included 5111 pregnant women with crown to rump length measurements in the first trimester (11 weeks 0 days to 13 weeks 6 days of gestation) and at least twice ultrasonic scanning records during the second and the third trimester. We excluded the pregnant women with unreliable last menstrual period (LMP) (*n* = 267), which was defined as (1) had used oral contraceptives during the year before pregnancy (*n* = 106), (2) had missing data on pre-pregnancy menstrual history (*n* = 68), or (3) the time interval between the gestational age estimated by date of LMP and crown to rump length (CRL) measurement was > 7 days (*n* = 93) [16]. Pregnant women who delivered a baby with a congenital malformation (*n* = 17) were additionally excluded. Of the remaining 4827 mother-child pairs, 3555 children attended the follow-up at age 2 years, and 2058 children who completed the neurodevelopment test at age 2 years were eligible for the final analysis.

### Fetal growth measurement

Fetal anthropometrics were measured by licensed sonographers to the nearest millimeter using standardized ultrasound procedures, and the data on fetal anthropometric measurements were extracted from sonographic notes in electronic health records. CRL was measured between 11 weeks 0 days to 13 weeks 6 days of gestation. Abdominal circumference, head circumference, and femur length were repeatedly measured in the second trimester (16 ± 2 and 23 ± 2 weeks of gestation) and the third trimester (30 ± 2, and 38 ± 2 weeks of gestation) by ultrasound scan. All of the ultrasound measurements were measured by certificated sonographers at the Department of Ultrasound Diagnosis in each study hospital following Chinese practice guidelines for obstetric ultrasound. All sonographers had obtained a Clinical Physicians’ and Doctors’ Qualification Certificate and a certificate of National Medical Equipment Use and had more than 3 years of experience performing fetal ultrasonography. The quality control group in the study hospitals implemented blind monthly re-examinations of a random 10% of scans to assure acceptable intra- and inter-observer variability. Estimated fetal weight (EFW) was calculated based on head circumference, abdominal circumference, and femur length, using the formula by Hadlock et al. [17]. Many studies have shown that in pregnant women with reliable LMP, gestational age can be calculated from LMP, so that first-trimester CRL can reflect first-trimester fetal size [18–22]. Therefore, we used CRL as an indicator for fetal size in the first trimester [19, 21]. Additionally, EFW is one of the most commonly used measures in prenatal care, reflecting comprehensive fetal growth status. It is also the key measure to diagnose the most common fetal complication, fetal growth restriction [23]. Consequently, EFW was used to indicate fetal size in the second and third trimester.

### Neurodevelopment assessment

The Bayley Scales of Infant Development (BSID) of China revision was used to evaluate child’s neurodevelopment at a mean age of 2.04 (SD: 0.08) years. BSID tests were conducted by licensed physicians at the study hospital, according to the standardized guidelines. BSID tests produce two main neurodevelopment scores: (1) mental development index (MDI), which represents cognitive, language, and personal/social development; (2) psychomotor development index (PDI), which represents motor ability, including fine and gross motor development. Raw scores of MDI and PDI were normalized into scores with a mean value of 100 points and a standardized deviation of 15 points.

### Covariates

Information on menstruation (including the date of LMP, menstrual regularity, and menstrual cycle duration), maternal sociodemographic characteristics (including maternal age at delivery, maternal education, annual family income, residence area, pre-pregnancy weight, and height), and lifestyle (including cigarette smoking, alcohol consumption, intake of folic supplement) were collected by a face-to-face interview. Parity, date of birth, child’s sex, and birth weight were derived from medical records. Gestational age was the interval time elapsed between the first day of LMP and the day of delivery. Maternal pre-pregnancy body mass index (BMI) was calculated by maternal pre-pregnancy weight (kg) divided by the squares of maternal height (m). Child weight and height at age 2 years were measured by well-trained staff when the children were followed up at the study hospital, with an accuracy of 0.01 kg and 0.1 cm, respectively. Directed acyclic graphs (DAG) were used to determine the confounding variables in our multiple adjustment analyses (Additional file 1: Fig S1). We considered maternal age at delivery (continuous), maternal education (≤ 12, > 12 years), annual family income (< 50,000, 50,000–100,000, > 100,000 yuan), residence area (central urban area, suburban area), pre-pregnancy BMI (underweight: < 18.5, normal: 18.5–24.0, overweight or obesity: ≥ 24.0 kg/m^2^), parity (nulliparous, parous), intake of folic acid supplement (yes, no), and child sex (male, female) as confounders in our study. A correlation matrix between covariates was conducted, and the results showed the correlations between covariates were weak (Additional file 1: Fig S2). Then, we applied variance inflation factors (VIF) to examine the multi-collinearity between variables. The results indicated no multi-collinearity between variables; thus, we included all of these variables in the models.

### Statistical analysis

The analytic plan for this study is briefly described in Additional file 1: Fig S3.

### Data preparation

Missing data (1528/8232, 18.6%) on EFW in the second and the third trimester were imputed using multiple imputations [24] by Markov chain Monte Carlo (MCMC) methods. The number of imputations was 20 times with relative efficiency > 0.97. To better reflect the uncertainty in the final result that arises from imputing the missing data, 20 datasets were created, and the results of the analyses of imputations were combined to generate valid statistical inferences. For confounding variables, the minority with missing data on continuous variables, such as pre-pregnancy BMI (*n* = 1), was imputed as the mean of the counterpart variable. Missing data on classified variables, including annual household income (*n* = 3) and intake of folic acid supplement (*n* = 1), were imputed as the mode of the counterpart variable.

### Main analysis

To improve the comparability of measured values of fetal size indicators in varied gestational ages, we standardized the CRL and EFW. We calculated the Z score for CRL according to an international standard proposed by the Intergrowth-21 study. However, the EFW standard proposed by Intergrowth-21 study does not cover the range before 22 weeks of gestation, so we calculated the Z score of EFW using the LMS (lambda, mu, sigma) methods [25]. Additional details are provided in Additional file 2: Supplementary Method [25–27]. We did scatter plots to observe the general relationship between the exposure (CRL and growth rate) and outcome (PDI and MDI) and applied generalized additive model to test the potential non-linear associations.

First, to investigate the association between first-trimester fetal size and childhood neurodevelopment, we applied general linear models to examine the linear association of first-trimester CRL Z score (as a continuous variable) with MDI and PDI at age 2 years. The CRL Z score was further divided into quartiles to evaluate the associations with MDI and PDI at age 2 years. *P* for linear trend of the association was assessed by entering the median value of each category of our measures as a continuous variable in the models.

Second, to examine whether later fetal growth was related to childhood neurodevelopment, we investigated the associations of second-trimester fetal size, third-trimester fetal size, and fetal growth rate with childhood neurodevelopment. We adjusted for CRL Z score in the model to check whether the association was independent of first-trimester fetal size. Generalized estimating equations with linear link were utilized to assess the associations of EFW Z score with MDI and PDI at age 2 years, considering that the EFW was repeatedly measured and correlated between the second and third trimester. Also, general linear models were applied to examine the association of fetal growth rate with neurodevelopment scores (MDI and PDI) at age 2 years. The fetal growth rate was the average EFW growth rate (change in Z score per week) from the second (16 ± 2 weeks) to the third trimester (38 ± 2 weeks). Fetal growth rate is defined as the change in fetal size between 2 time points during gestation [28, 29]. To standardize comparison of growth rate, the change in fetal size between ultrasounds was divided by the exact number of weeks between examinations, to create a change per week [30]. To be specific, fetal growth rate = (EFW Z score at third trimester − EFW Z score at second trimester)/(gestational age at third trimester − gestational age at second trimester). Fetal growth rate was divided into quartiles to fit the models to evaluate the associations with neurodevelopment scores (MDI and PDI) at age 2 years.

Third, we conducted a joint analysis of first-trimester fetal size and fetal growth patterns to explore whether there are differences in the association between fetal growth patterns and childhood neurodevelopment in different first-trimester fetal size strata. We investigated the effect of different fetal growth patterns on neurodevelopment in children with different first-trimester fetal sizes. We divided our population into three strata based on the tertiles of the first-trimester CRL Z score. The highest, middle, and lowest tertile of CRL Z score were defined as “optimal first-trimester fetal size”, “median first-trimester fetal size”, and “restricted first-trimester fetal size”, respectively. In each strata, the population was further divided into three growth patterns based on the highest, middle, and lowest tertile of fetal growth rate, which was defined as “intrauterine accelerated growth” (IAG), “intrauterine median growth” (IMG), and “intrauterine faltering growth” (IFG), respectively. In each CRL strata, general linear models were used to examine the association of different fetal growth patterns with neurodevelopment scores (MDI and PDI).

### Sensitivity analysis

To better explain the associations of first-trimester fetal size, fetal growth rate, and fetal growth patterns with childhood neurodevelopment and to test their robustness, we did several sensitivity analyses. In order to test whether study associations were influenced by potentially pathology-related growth, extreme growth, or birth outcomes, we (1) excluded mothers with gestational diabetes mellitus (*n* = 172) and pregnancy-induced hypertension (*n* = 43); (2) excluded children with preterm birth (*n* = 54), low birth weight (< 2500 g) (*n* = 35), and macrosomia (birth weight ≥ 4000 g) (*n* = 108); (3) additionally adjusted for birth weight and gestational age at delivery (*n* = 2058) in the models. Moreover, to investigate whether the study associations were independent of postnatal growth, we additionally adjusted for the BMI Z scores at age 2 years (*n* = 1877), which were calculated based on the World Health Organization growth charts for children [31].

To rule out the potential information bias on gestational age at CRL measurement, which is caused by the menstrual cycle, we did sensitivity analyses by restricting the population to participants who had regular menstrual cycles (*n* = 1885). In addition, two additional sensitivity analyses by restricting the population to participants with a menstrual cycle of 28 plus or minus 3 days (*n* = 1842) and additionally adjusting for menstrual cycle duration (*n* = 2025) were performed. In addition, to reduce the potential selection bias caused by differences in basic characteristics of the included and excluded populations, we applied inverse probability weighting [32, 33] based on the distribution of basic characteristics in the included participants and the whole cohort.

### Supplementary analysis

A supplementary analysis of univariate and multivariate analyses was conducted to clarify the individual role of the covariates on childhood neurodevelopment. To identify potential effect modification of factors including fetal sex, pre-pregnancy BMI, and maternal age at delivery, we conducted stratified analyses as well as interaction tests for these factors.

Statistical analyses were conducted by SAS 9.4 except for the calculation of Z score of fetal size indicators, which was conducted by R 4.0.3 using “GAMLSS” packages. *P* values were calculated with the Wald *t* test, and *P* < 0.05 in the two-sided tests was defined as significant.

## Results

### Characteristics of participants

Basic characteristics of the 2058 mother-child pairs are depicted in Table 1. The mean (SD) age of mothers at delivery was 29.0 (3.5) years. Fetal CRL was measured at 12.5 (SD, 0.6) weeks of gestation. The mean (SD) of gestation duration was 39.34 (1.12) weeks. Neurodevelopment was tested at 2.0 (SD, 0.1) years. The mean (SD) of MDI and PDI was 108.80 (21.74) and 107.75 (17.98), respectively. As shown in Additional file 3: Table S1, the first-trimester CRL of fetuses included in the present study was comparable to those excluded. However, in this study, the proportion of participants with more than 12 years of education, annual household income of 50,000 to 100,000 yuan, and being nulliparous was marginally higher, while the proportion of participants living in the central urban area was slightly lower.

**Table 1:** Basic characteristics of the study population (*n* = 2058). Values are numbers (percentages) unless stated otherwise

Characteristics	No. (%)
Maternal	
Mean (SD), age, years	28.95 (3.50)
Education levels	
≤ 12 years	372 (18.08)
> 12 years	1686 (81.92)
Annual family income	
< 50,000 yuan	246 (11.95)
50,000–100,000 yuan	750 (36.44)
> 100,000 yuan	1062 (51.60)
Residence area	
Suburban area	419 (20.36)
Central urban area	1639 (79.64)
Pre-pregnancy body mass index	
Underweight (< 18.5 kg/m^2^)	374 (18.17)
Normal weight (18.5–24.0 kg/m^2^)	1384 (67.25)
Overweight or obesity (≥ 24 kg/m^2^)	300 (14.58)
Parity	
Nulliparous women	1705 (82.85)
Parous women	353 (17.15)
Intake of folic acid supplement	
No	652 (31.68)
Yes	1406 (68.32)
Median (IQR), menstrual cycle duration	29.89 (2.00)
Menstrual cycle duration within 25–31 days	1842 (89.50)
Menstrual cycle duration < 25 days or > 31 days	216 (10.50)
Fetal and birth	
Mean (SD), gestational age at first-trimester crown to rump length measurement, weeks	12.52 (0.58)
Mean (SD), first-trimester crown to rump, cm	6.20 (0.79)
Mean (SD), gestational age at second-trimester estimated fetal weight measurement, weeks	24.27 (0.55)
Mean (SD), second-trimester estimated fetal weight, g	674,90 (71.06)
Mean (SD), gestational age at third-trimester estimated fetal weight measurement, weeks	37.41 (0.81)
Mean (SD), third-trimester estimated fetal weight, g	2947 (281.89)
Mean (SD), gestation duration, weeks	39.34 (1.12)
Mean (SD), birth weight, g	3332.24 (416.53)
Sex	
Male	1055 (51.26)
Female	1003 (48.74)
Childhood	
Neurodevelopmental assessment	
Mean (SD), age at the neurodevelopment assessment, months	2.04 (0.08)
Mean (SD), MDI at the age of 2 years, score	108.80 (21.74)
Mean (SD), PDI at the age of 2 years, score	107.75 (17.98)

Missing data on maternal pre-pregnancy BMI (*n* = 1) was imputed as the mean of the counterpart variable; missing data on annual household income (*n* = 3) and intake of folic acid supplement (*n* = 1) were imputed as the mode of the counterpart variable

*Abbreviations*: *MDI* Mental development index, *PDI* Psychomotor development index

### First-trimester fetal size and neurodevelopment

The scatter plots (Additional file 1: Fig S4) and the non-linear test results (Additional file 3: Table S2) indicated no significant non-linear relationship exists between the CRL Z score and neurodevelopment scores (PDI and MDI). We observed significant associations of first-trimester CRL with neurodevelopment scores at age 2 years (Table 2). Full adjustment of confounders did not weaken the associations. Specifically, each Z score increment in CRL was associated with a higher score in MDI (adjusted beta estimate = 1.19, (95% CI: 0.13, 2.25), *P* = 0.03) and PDI (adjusted beta estimate = 1.36, (95% CI: 0.46, 2.26), *P* < 0.01) at age 2 years. Consistent associations were observed in the quartile CRL models with a significant linear trend (MDI: *P* for trend = 0.03; PDI: *P* for trend < 0.01). Compared with the lowest quartile, children in the highest quartile of CRL had higher scores of MDI (adjusted beta estimate = 2.88, (95% CI: 0.27, 5.49), *P* = 0.03) and PDI (adjusted beta estimate = 3.88, (95% CI: 1.68, 6.08), *P* < 0.01).

**Table 2:** First-trimester fetal size in relation to neurodevelopment scores at age 2 years (*n* = 2058)^a^

CRL	MDI, *β* (95% CI)		PDI, *β* (95% CI)	
	Unadjusted	Adjusted^a^	Unadjusted	Adjusted^a^
Continuous				
Per increment of Z scores	1.09 (0.01, 2.16)*	1.19 (0.13, 2.25)*	1.45 (0.56, 2.34)*	1.36 (0.46, 2.26)*
Categorized^b^				
Quartile 1	Reference	Reference	Reference	Reference
Quartile 2	1.88 (− 0.75, 4.52)	1.66 (− 0.92, 4.23)	2.57 (0.40, 4.75)*	2.38 (0.20, 4.55)*
Quartile 3	1.61 (− 1.07, 4.29)	1.72 (− 0.90, 4.35)	3.45 (1.23, 5.66)*	3.31 (1.10, 5.53)*
Quartile 4	2.64 (− 0.01, 5.29)	2.88 (0.27, 5.49)*	4.09 (1.91, 6.28)*	3.88 (1.68, 6.08)*
*P* for trend	0.07	0.03	< 0.001	< 0.001

Values are regression coefficients (95% confidence interval) estimated by general linear models. *P*-values were calculated with the Wald *t* test

*Abbreviations*: *MDI* Mental development index, *PDI *Psychomotor development index, *CI *Confidence interval, *CRL *Crown to rump length

^a^Adjusted for maternal age, maternal education, annual household income, residence area, pre-pregnancy body mass index, parity, intake of folic acid supplement, and child’s sex

^b^CRL (Z scores): quartile 1: <  − 0.71; quartile 2: − 0.71 to − 0.01; quartile 3: − 0.01 to 0.69; quartile 4: > 0.69

^*^P < 0.05

### Later fetal growth and neurodevelopment

For second-trimester fetal size, which was indicated by EFW, each Z score increment in second-trimester EFW was associated with a higher score in MDI (adjusted beta estimate = 0.81, (95% CI: 0.34, 1.27), *P* < 0.01) and PDI (adjusted beta estimate = 0.85, (95% CI: 0.45, 1.24), *P* < 0.01) at age 2 years. However, the association between second-trimester EFW and neurodevelopment was insignificant when additionally adjusted for first-trimester CRL Z score (Additional file 3: Table S3). After adjusting for first-trimester CRL Z score, the association between third-trimester fetal size (presented by EFW) and MDI remained significant (adjusted beta estimate = 0.97, (95% CI: 0.50, 1.45), *P* < 0.001), while the association between third-trimester fetal size and PDI became boundary significant (adjusted beta estimate = 0.41, (95% CI: 0.01, 0.82), *P* = 0.05).

The scatter plots (Additional file 1: Fig S4) and the non-linear test results (Additional file 3: Table S2) indicated no significant non-linear relationship exists between the feta growth rate and neurodevelopment scores (PDI and MDI). We found that fetal growth rate (the average EFW growth rate from the second to the third trimester) was associated with higher scores in MDI at age 2 years (Table 3). After full adjustment of confounders, per unit (a Z score per week) increment in fetal growth rate was associated with a higher score in MDI at age 2 years (adjusted beta estimate = 17.22, (95% CI: 7.87, 26.57), *P* < 0.001). Consistent associations were observed in the quartile models (4th quartile vs. the 1st quartile: adjusted beta estimate = 3.28, (95% CI: 1.72, 4.83), *P* < 0.001) with a significant linear trend between fetal growth rate and MDI (*P* for trend < 0.001). No significant association was observed between fetal growth rate and PDI at age 2 years.

**Table 3:** Fetal growth rate in relation to neurodevelopment scores at age 2 years (*n* = 2058)^a^

Fetal growth rate^a^	MDI, *β* (95% CI)		PDI, *β* (95% CI)	
	Unadjusted	Adjusted^b^	Unadjusted	Adjusted^c^
Continuous				
Per increment of EFW Z scores/week	14.86 (5.61, 24.11)*	17.22 (7.87, 26.57)*	6.79 (− 1.16, 14.75)	4.64 (− 3.41, 12.68)
Categorized^c^				
Quartile 1	Reference	Reference	Reference	Reference
Quartile 2	2.58 (0.86, 4.31)*	2.52 (0.80, 4.24)*	0.72 (− 0.64, 2.08)	0.55 (− 0.80, 1.91)
Quartile 3	2.19 (0.56, 3.82)*	2.20 (0.58, 3.83)*	0.12 (− 1.23, 1.47)	− 0.02 (− 1.38, 1.33)
Quartile 4	2.87 (1.33, 4.40)*	3.28 (1.72, 4.83)*	1.15 (− 0.15, 2.45)	0.86 (− 0.45, 2.17)
*P* for trend	< 0.001	< 0.001	0.16	0.32

Values are regression coefficients (95% confidence interval) estimated by general linear models based on multiply imputed data. *P*-values were calculated with the Wald *t* test

*Abbreviations*: *MDI *Mental development index, *PDI *Psychomotor development index, *CI *Confidence interval, *EFW *Estimated fetal weight

^a^Fetal growth rate = (EFW Z scores at (38 ± 2 weeks) − EFW Z scores at (16 ± 2 weeks))/(gestational age at (38 ± 2 weeks) − gestational age at (16 ± 2 weeks))

^b^Adjusted for maternal age, maternal education, annual household income, residence area, pre-pregnancy body mass index, parity, intake of folic acid supplement, child’s sex, and CRL Z scores

^c^Fetal growth rate (EFW Z scores/week): quartile 1: <  − 0.04; quartile 2: − 0.04 to − 0.00; quartile 3: − 0.00 to 0.04; quartile 4: > 0.04

^*^P < 0.05

### Joint analysis of CRL and fetal growth patterns

**Fig. 1:**
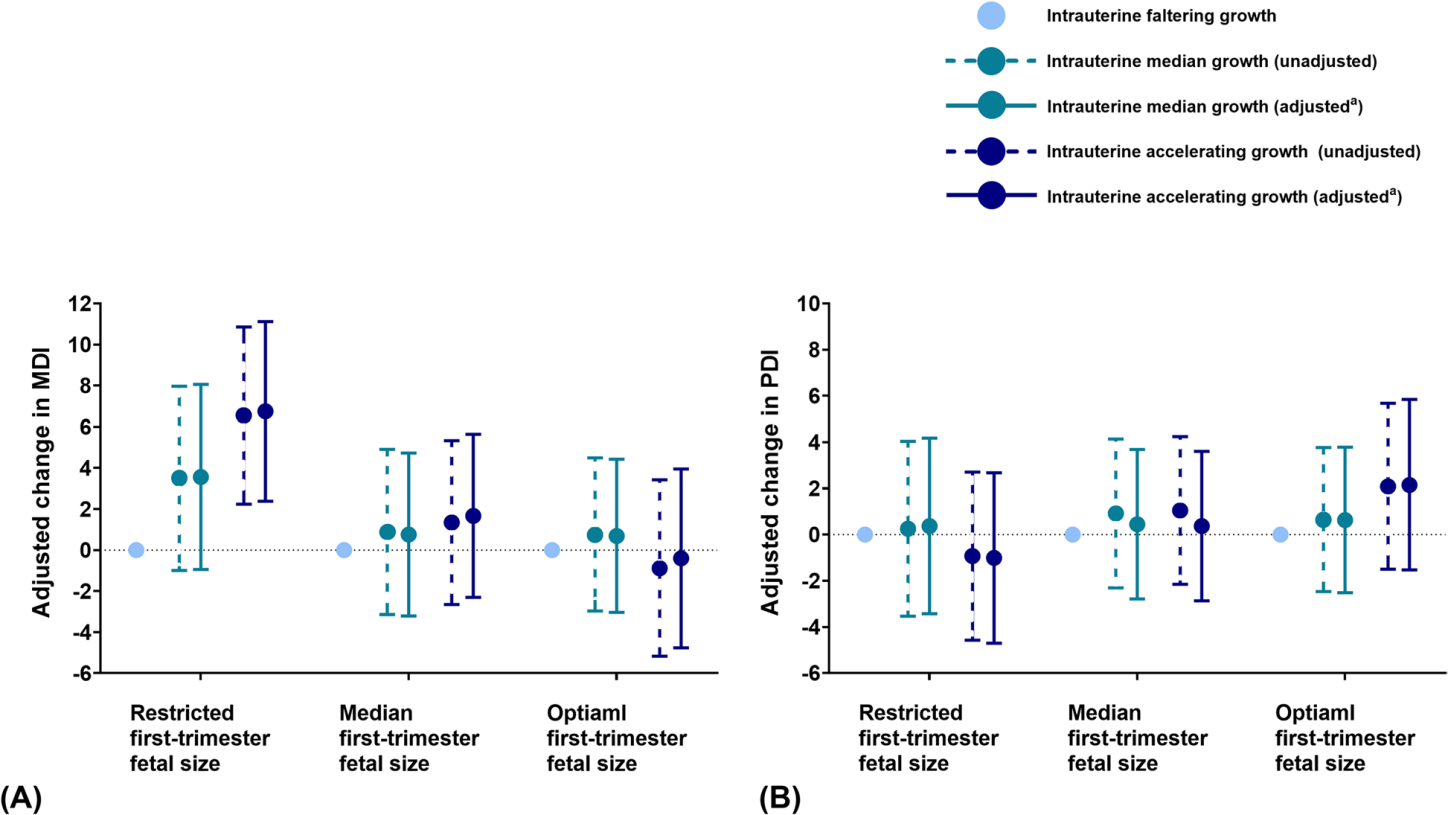
Joint analysis of CRL^a^ and fetal growth patterns^b^ in relation to childhood neurodevelopment. **A** Joint analysis of CRL and fetal growth patterns of fetal growth patterns in relation to MDI. **B** Joint analysis of CRL and fetal growth patterns of fetal growth patterns in relation to PDI. *Abbreviations*: *MDI*, mental development index; *PDI*, psychomotor development index; *CRL*, crown to rump length. Values are regression coefficients (95% confidence interval) estimated by general linear models based on multiple imputed data. ^a^ Adjusted for maternal age, maternal education, annual household income, residence area, pre-pregnancy body mass index, parity, intake of folic acid supplement, child’s sex, and CRL Z score. ^b^ Restricted first-trimester fetal size: the lowest tertile of CRL Z score (< − 0.38); median first-trimester fetal size: the middle tertile of CRL Z score (− 0.38 to 0.36); optimal first-trimester fetal size: the highest tertile of CRL Z score (> 0.36). ^c^ Intrauterine faltering growth: the lowest tertile of fetal growth rate (< − 0.022 EFW Z score/week); intrauterine median growth: the middle tertile of fetal growth rate (− 0.022 to 0.023 EFW Z score/week); intrauterine accelerated growth: the highest tertile of fetal growth rate (> 0.023 EFW Z score/week)

The IAG pattern was associated with higher MDI scores compared to the IFG pattern, and this association was most pronounced in children with restricted first-trimester fetal size. In the strata of “restricted first-trimester fetal size” (the lowest tertile of first-trimester CRL), children with IMG pattern and IAG pattern had higher MDI scores (IMG: adjusted beta estimate = 4.10, (95% CI: 1.40, 6.81), *P* < 0.001; IAG: adjusted beta estimate = 6.14, (95% CI: 3.80, 8.49), *P* < 0.001) compared to children with IFG pattern (Fig. 1A, detailed estimates, and 95% CIs are shown in Additional file 3: Table S4). Nevertheless, no significant difference in PDI scores was observed among fetal growth patterns in any of CRL strata. In the strata of “restricted first-trimester fetal size”, children with IAG pattern had an indistinguishable score in PDI (adjusted beta estimate = − 0.85, (95% CI: − 3.12, 1.42), *P* = 0.46) compared to children with IFG pattern (Fig. 1B, detailed estimates, and 95% CIs are shown in Additional file 3: Table S4).

### Results of sensitivity analysis

The results of sensitivity analyses are depicted in Additional file 3: Table S5. The results were robust when we (1) excluded pregnant women with pregnancy-induced hypertension and gestational diabetes mellitus; (2) excluded children with low birth weight, macrosomia, and preterm; and (3) additionally adjusted for birth weight and gestational age at delivery. When we adjusted for BMI Z scores at age 2 years, the estimates did not substantially change.

The effect estimates for neurodevelopment scores did not materially change with a restriction to participants who had a self-reported regular menstrual cycle, a restriction to participants with menstrual cycles of 28 plus or minus 3 days, and with additional adjustment for menstrual cycle duration. After applying inverse probability weighting, the distribution of basic characteristics between included and excluded mother-child pairs was comparable (Additional file 3: Table S6), and the associations of first-trimester fetal size and fetal growth rate with neurodevelopment scores did not weaken (Additional file 3: Table S5).

### Results of supplementary analysis

Results of univariate analyses and multivariate analyses for childhood neurodevelopment are presented in Additional file 3: Table S7. In the multivariate analysis that included maternal characteristics and child sex, we observed that higher maternal education, higher annual family income, intake of folic acid supplement during the first trimester, female children, and gestational age at delivery were significantly and positively associated with MDI scores. Among the associations of the variables with PDI, we observed a significant positive association of parous women, and gestational age at delivery with PDI scores, while pregnancy-induced hypertension was significantly negatively associated with PDI scores.

Stratified analyses and interaction tests for fetal sex, pre-pregnancy BMI, and maternal age at delivery showed that these factors did not significantly modify the effect of the study association (Additional file 3: Table S8).

## Discussion

For the first time, this study provides epidemiological evidence linking restricted first-trimester fetal size to mental and psychomotor developmental delay in early childhood. Growth rate in the later fetal stage was a positive predictor for mental development but not psychomotor development at age 2 years. For children with restricted first-trimester fetal size, the intrauterine accelerated growth was an advantage for their mental development but not psychomotor development, indicating the early fetal stage is a critical time window for children’s psychomotor development, whereas the early fetal stage and late fetal stage are both critical for children mental development.

A previous study that assessed associations between fetal size at early pregnancy (14 weeks of gestation) and academic attainment in 6995 children aged 6 to 7 years, reported that children with increased fetal size at early pregnancy (approximately 14 weeks) had higher odds of achieving the expected standard in mathematics, science, reading, and writing [11]. However, this study estimated the early pregnancy fetal size by models rather than using actual measured values, requiring the use of more reliable measurement strategies to repeat the results. Previous studies have shown that fetal sizes in the second and third trimester were associated with children’s neurodevelopment. However, it is hard to distinguish whether the observed associations were biased by the baseline first-trimester fetal size [8, 10]. We observed that the positive associations between second-trimester fetal size and neurodevelopment were insignificant when additionally adjusting for first-trimester CRL, indicating that the unequal first-trimester fetal size may contribute to these associations.

In this study, we observed that restricted first-trimester fetal size was a risk factor for mental developmental delay in early childhood. However, a higher fetal growth rate during the second and third trimesters was beneficial for early childhood mental development, providing a second chance for fetuses with restricted first-trimester fetal size to catch up with mental neurodevelopment. In the joint analysis of first-trimester fetal size and fetal growth rate, we defined intrauterine growth patterns by fetal growth rate (the average EFW growth rate from the second and the third trimester) rather than simply comparing fetal size in early or late pregnancy. In this way, we considered fetal size at different gestational periods and also reduced the impact on the study associations by possible misclassification of fetal growth patterns due to measurement errors in the first trimester (i.e., fetuses with measurement errors towards smaller first-trimester size would seem to be “IAG”). In keeping with the results, fetuses with the IAG pattern had significantly improved mental development scores compared with those with the IFG pattern, particularly among fetuses with restricted (the lowest tertile) first-trimester fetal size. The restricted first-trimester fetal size was associated with psychomotor developmental delay, but a higher fetal growth rate or IAG pattern did not improve psychomotor development, suggesting the effects of restricted first-trimester growth on psychomotor development are long-term and are hard to compensate by intrauterine accelerated growth in later fetal stages.

From the results of several sensitivity analyses, our study associations were robust. Firstly, we excluded factors that may influence later fetal growth (e.g., gestational diabetes mellitus, low birth weight, etc.) and additionally adjusted for birth weight and gestational age at delivery in the models, and the results suggested that the associations were less affected by pathology-related growth and extreme growth as well as birth outcomes. Secondly, we also examined the role of postnatal growth in the study association, and the results suggested that the associations are independent of postnatal growth. Thirdly, we examined the effect of potential information bias on gestational age caused by the menstrual cycle on the study associations, and the results suggested that the effect was limited. Finally, we also considered the influence of that potential selection bias due to differences in the basic characteristics of the included and excluded populations on the study association. After applying inverse probability weighting, the results did not change materially, which indicates the associations we observed were unlikely to be due to the potential selection bias of our population. These results showed that it is less likely that our findings were the result of faulty inferences based on methodology limitations.

The embryo’s brain and nervous system begin to develop in the third week of gestation [4]. Brain cells proliferate rapidly in the first trimester [7]. It is also the critical period for neural tube development when the earliest nervous tissue eventually develops into the brain and spinal cord [5]. As the first trimester is the period to build a basic neural framework, it is not hard to explain why the first trimester is critical for neurodevelopment. In addition, we observed that fetal growth after the first trimester, especially the third trimester is also critical for mental development. The underlying mechanism may involve patterns of brain development. Development of the brain starts with the most basic systems, such as the brainstem, spinal cord, sensory-motor regions, and visual and auditory systems [4]. The function of higher-level perceptual, cognitive, and emotional capabilities needs to be built upon basic neural circuitry and thus develop later [34]. Arborization, synaptogenesis, neuronal migration, and myelination accelerate during the third trimester. These events regulate the development of neural circuits and increase connectivity, making the brain more sophisticated and complex [6]. Additionally, substantial increases in brain volume, brain weight, and gyral and sulcal development happened during late pregnancy [35], which was associated with better neurodevelopment [36].

The major strength of this study was that fetal size was assessed longitudinally and systematically. Information on ultrasound-measured fetal anthropometrics during different fetal stages allowed us to explore the critical intrauterine windows associated with neurodevelopment. Furthermore, detailed information on the participants from the prospective cohort allowed us to adjust for multiple confounders. However, some limitations had to be acknowledged in our study. First, we acknowledged that, as with all observational studies, measurement errors could not be completely eliminated in this study, although all the sonographers followed a standardized procedure when performing ultrasound measurements and the routine blind tests showed that measurement errors were not significant across sonographers. Second, we restricted the study population to those with reliable LMP to ensure that the gestational age obtained was more accurate. We also performed several sensitivity analyses, which showed that the menstrual cycle had a limited effect on the study association by affecting gestational age. However, we could not completely rule out the misclassification of gestational age. Third, given that this study was conducted among a single-center birth cohort and the characteristics of the population may differ from the general population (e.g., lower prevalence of preterm and no participant had preexisting diabetes or hypertension). Also, this study did not gather data on miscarriages and stillbirths, it may lead to biased results because of competing risk given that small CRL may be related to subsequent miscarriage. Even though we applied inverse probability weighting to reduce the influence of potential selection bias of our population on the associations, the effect of unmeasured factors cannot be excluded. Further research should investigate the associations in multi-center populations and different ethnic groups or use other measures of fetal brain development to test the generalizability of our results. Currently, care in early pregnancy is still undervalued. Our study proposes a positive association between first-trimester fetal size and childhood neurodevelopment, which was not the result of a few pathology-related or extreme growths. These findings may contribute to the earlier identification of children at risk of neurodevelopmental delay and provide clues on the timing of appropriate interventions to improve neurodevelopment. Future studies should validate our results in diverse populations and further investigate the factors affecting fetal size in early pregnancy to explore possible interventions.

## Conclusions

In this prospective longitudinal study, restricted first-trimester fetal size was related to mental and psychomotor developmental delay in early childhood. Increased fetal growth rate, as well as intrauterine accelerated growth pattern after the first trimester, was associated with improved mental development but not psychomotor development. The above associations were independent of postnatal growth. These findings suggest that the early fetal stage is a key period for both mental and psychomotor development; the association between restricted first-trimester fetal size with psychomotor developmental delay might not be compensated by later intrauterine accelerated growth. This study provides clues on the timing of appropriate interventions to improve neurodevelopment in early childhood.

### Supplementary Information

**Supplementary Material 1.**

**Supplementary Material 2.**

**Supplementary Material 3.**

## Data Availability

The datasets analyzed during the current study are not publicly available due to ethical restrictions but are available from the corresponding author on reasonable request.

## Abbreviations

BMI: Body mass index
BSID: Bayley scales of infant development
CRL: Crown to rump length
DAG: Directed acyclic graphs
EFW: Estimated fetal weight
IAG: Intrauterine accelerated growth
IFG: Intrauterine faltering growth
IMG: Intrauterine median growth
LMP: Last menstrual period
LMS: Lambda, mu, sigma
MCMC: Markov chain Monte Carlo
MDI: Mental developmental index
PDI: Psychomotor developmental index
VIF: Variance inflation factors
