# Supplementary material for: First-trimester fetal size, accelerated growth in utero, and child neurodevelopment in a cohort study
Source: BMC Med. 2024 Apr 29;22:181. doi: 10.1186/s12916-024-03390-3 (PMC11059611; doi:10.1186/s12916-024-03390-3)
Supplement: Supplementary file 1 — Supplementary Material 1. [file 12916_2024_3390_MOESM1_ESM.docx]

**Fig S1.** DAG defined covariates in multiple adjustment analyses of fetal growth with childhood neurodevelopment at age two years.


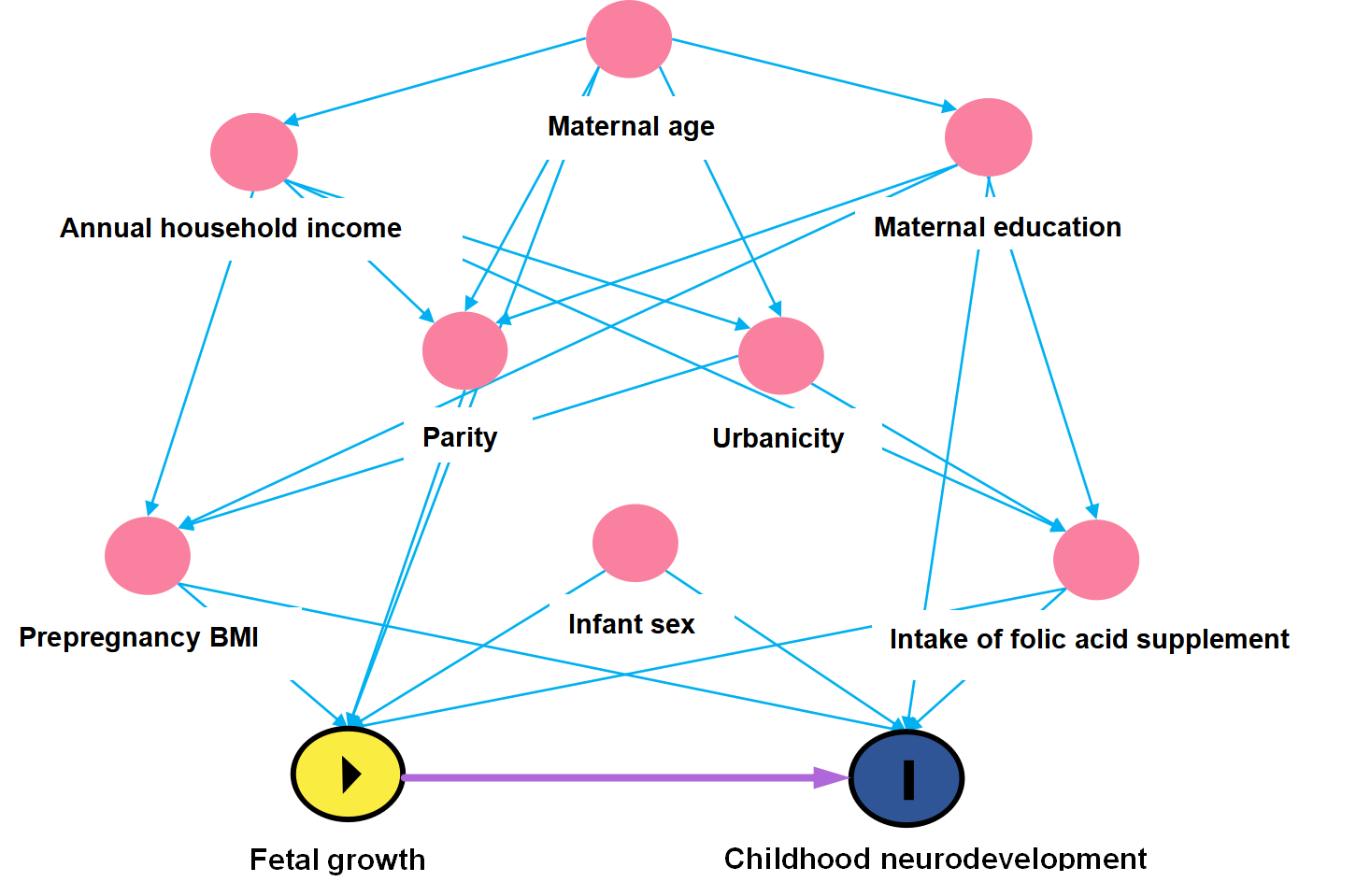


**Fig S2.** Heap map of the correlation matrix between covariates.


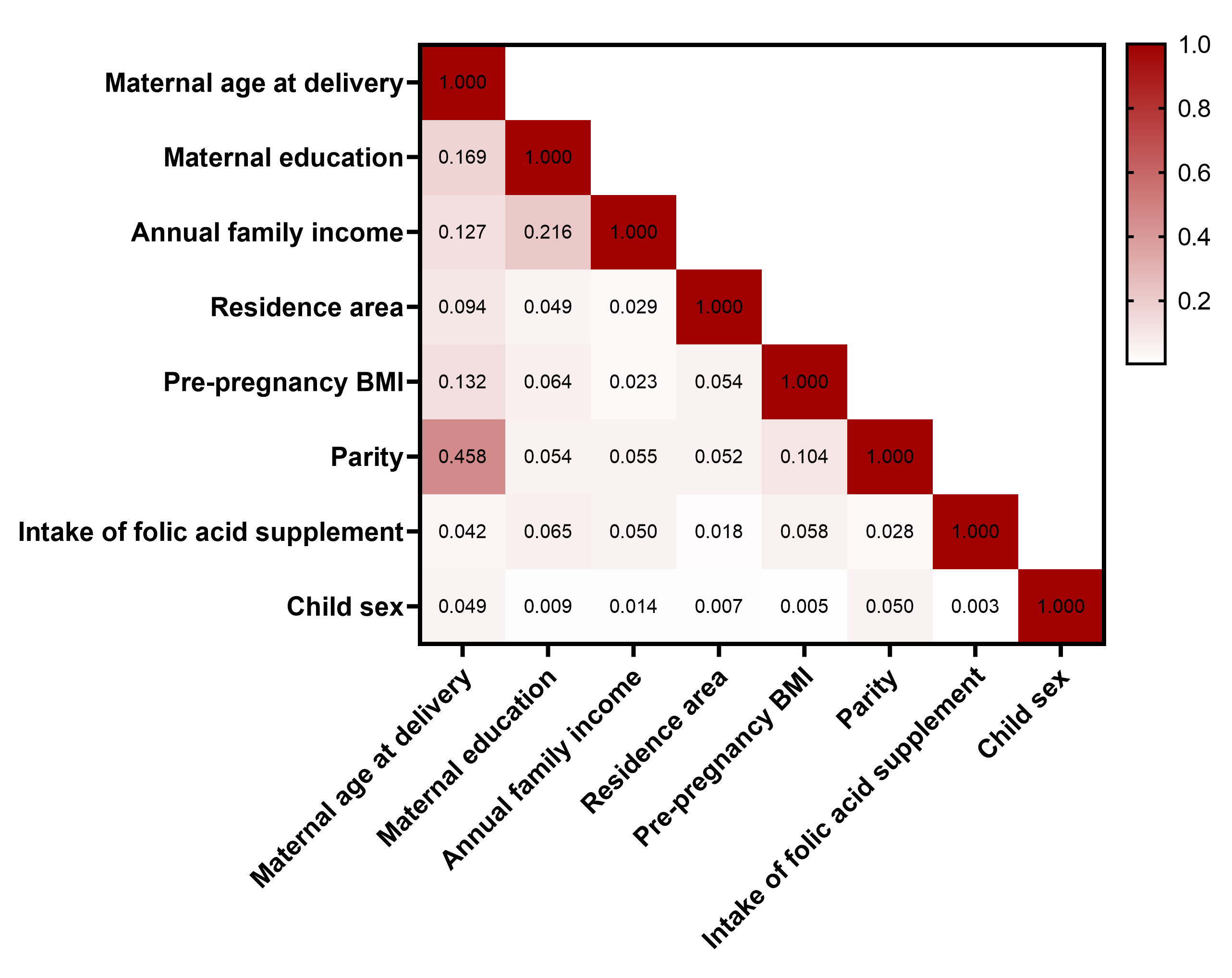


Values are correlation coefficients from Cramer's V which is a measure of the strength of association between two nominal variables.

**Fig S3.** The analytic plan for this study.


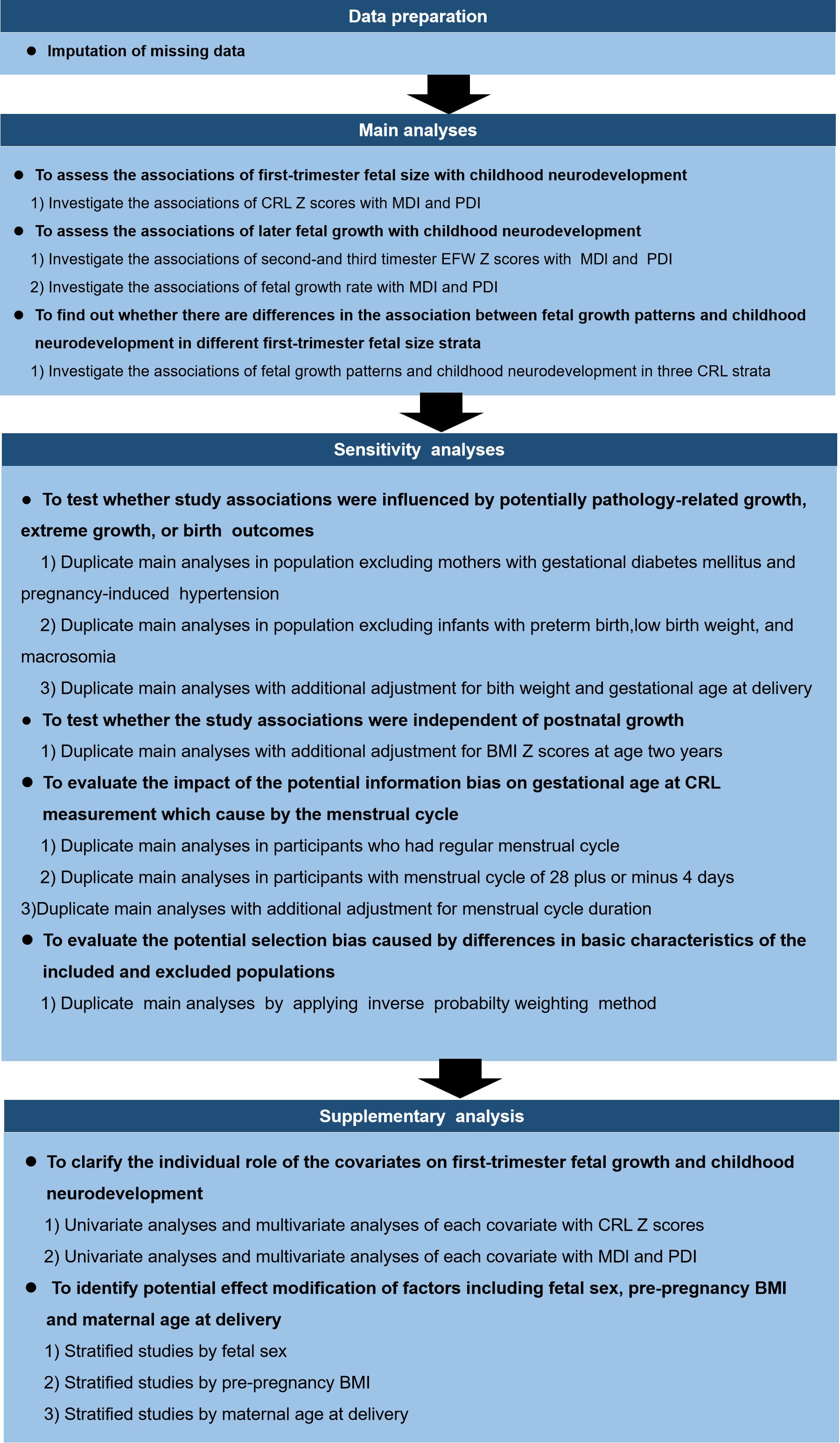


**Fig S4.** The scatter plot of CRL Z score with PDI and MDI.

**
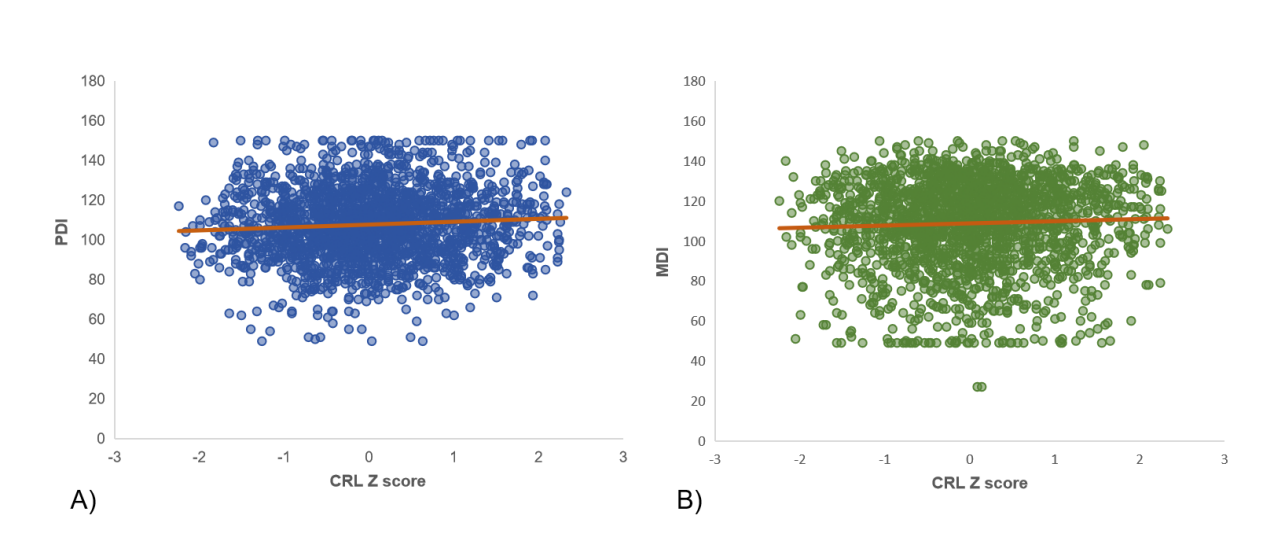
**
