# Supplementary material for: First-trimester fetal size, accelerated growth in utero, and child neurodevelopment in a cohort study
Source: BMC Med. 2024 Apr 29;22:181. doi: 10.1186/s12916-024-03390-3 (PMC11059611; doi:10.1186/s12916-024-03390-3)
Supplement: Supplementary file 2 — Supplementary Material 2. [file 12916_2024_3390_MOESM2_ESM.docx]

**Supplementary Method**

The Z score of CRL according to an international standard proposed by the Intergrowth-21 study (Table 1).[1] However, the EFW standard proposed by Intergrowth-21 study do not cover the range before 22 weeks of gestation, so we calculated the Z score of EFW using the LMS (lambda, mu, sigma) methods.[2] Briefly, the measured values were transformed to normality by Box-Cox transformation and were modeled against a cubic spline of gestational age based on different distribution families provided by the ‘GAMLSS’ package.[3] Distribution includes Box-Cox Cole and Green distribution (BCCG), Box-Cox power exponential distribution (BCPE), and Box-Cox-t distribution (BCT). BCCG distribution estimates three parameters (the median, coefficient of variation (mu), and a Box-Cox power transformation at each gestational age to remove skewness (lambda), thereby making the data roughly normally distributed). BCT distribution assumes a shifted and scaled (truncated) t distribution to take account of skewness and leptokurtosis. BCPE distribution assumes a Box-Cox power exponential distribution to take account of skewness, platykurtosis, and leptokurtosis. According to Akaike's information criterion, the best-fitting models were selected to calculate gestational-age-adjusted Z score for all of the fetal size indicators.

**Table 1.** International fetal size standards in the first trimester.

| **Gestational age (days)** | **Z scores** | | | | | | |
| --- | --- | --- | --- | --- | --- | --- | --- |
|  | **-3** | **-2** | **-1** | **0** | **1** | **2** | **3** |
| 58 | 4.1 | 7.6 | 11.1 | 14.6 | 18.1 | 21.6 | 25.1 |
| 59 | 5.3 | 8.9 | 12.5 | 16.1 | 19.7 | 23.3 | 26.9 |
| 60 | 6.4 | 10.1 | 13.8 | 17.5 | 21.2 | 24.9 | 28.6 |
| 61 | 7.6 | 11.4 | 15.2 | 19.0 | 22.8 | 26.6 | 30.4 |
| 62 | 8.8 | 12.7 | 16.6 | 20.5 | 24.3 | 28.2 | 32.1 |
| 63 | 10.0 | 14.0 | 18.0 | 21.9 | 25.9 | 29.9 | 33.9 |
| 64 | 11.2 | 15.3 | 19.4 | 23.4 | 27.5 | 31.6 | 35.7 |
| 65 | 12.4 | 16.6 | 20.8 | 24.9 | 29.1 | 33.3 | 37.5 |
| 66 | 13.6 | 17.9 | 22.2 | 26.5 | 30.7 | 35.0 | 39.3 |
| 67 | 14.8 | 19.2 | 23.6 | 28.0 | 32.4 | 36.8 | 41.1 |
| 68 | 16.1 | 20.6 | 25.0 | 29.5 | 34.0 | 38.5 | 43.0 |
| 69 | 17.3 | 21.9 | 26.5 | 31.1 | 35.7 | 40.2 | 44.8 |
| 70 | 18.6 | 23.3 | 28.0 | 32.6 | 37.3 | 42.0 | 46.7 |
| 71 | 19.9 | 24.6 | 29.4 | 34.2 | 39.0 | 43.8 | 48.5 |
| 72 | 21.2 | 26.0 | 30.9 | 35.8 | 40.7 | 45.5 | 50.4 |
| 73 | 22.5 | 27.4 | 32.4 | 37.4 | 42.3 | 47.3 | 52.3 |
| 74 | 23.8 | 28.8 | 33.9 | 39.0 | 44.0 | 49.1 | 54.2 |
| 75 | 25.1 | 30.2 | 35.4 | 40.6 | 45.8 | 50.9 | 56.1 |
| 76 | 26.4 | 31.7 | 36.9 | 42.2 | 47.5 | 52.7 | 58.0 |
| 77 | 27.7 | 33.1 | 38.5 | 43.8 | 49.2 | 54.6 | 59.9 |
| 78 | 29.1 | 34.6 | 40.0 | 45.5 | 51.0 | 56.4 | 61.9 |
| 79 | 30.5 | 36.0 | 41.6 | 47.1 | 52.7 | 58.3 | 63.8 |
| 80 | 31.8 | 37.5 | 43.1 | 48.8 | 54.5 | 60.1 | 65.8 |
| 81 | 33.2 | 39.0 | 44.7 | 50.5 | 56.3 | 62.0 | 67.8 |
| 82 | 34.6 | 40.5 | 46.3 | 52.2 | 58.0 | 63.9 | 69.8 |
| 83 | 36.0 | 42.0 | 47.9 | 53.9 | 59.8 | 65.8 | 71.8 |
| 84 | 37.4 | 43.5 | 49.5 | 55.6 | 61.6 | 67.7 | 73.8 |
| 85 | 38.8 | 45.0 | 51.1 | 57.3 | 63.5 | 69.6 | 75.8 |
| 86 | 40.3 | 46.5 | 52.8 | 59.0 | 65.3 | 71.5 | 77.8 |
| 87 | 41.7 | 48.1 | 54.4 | 60.8 | 67.1 | 73.5 | 79.8 |
| 88 | 43.2 | 49.6 | 56.1 | 62.5 | 69.0 | 75.4 | 81.9 |
| 89 | 44.6 | 51.2 | 57.7 | 64.3 | 70.8 | 77.4 | 83.0 |
| 90 | 46.1 | 52.8 | 59.4 | 66.1 | 72.7 | 79.4 | 86.0 |
| 91 | 47.6 | 54.4 | 61.1 | 67.8 | 74.6 | 81.3 | 88.1 |
| 92 | 49.1 | 56.0 | 62.8 | 69.6 | 76.5 | 83.3 | 90.2 |
| 93 | 50.6 | 57.6 | 64.5 | 71.4 | 78.4 | 85.3 | 92.3 |
| 94 | 52.1 | 59.2 | 66.2 | 73.3 | 80.3 | 87.3 | 94.4 |
| 95 | 53.7 | 60.8 | 68.0 | 75.1 | 82.2 | 89.4 | 96.5 |
| 96 | 55.2 | 62.5 | 69.7 | 76.9 | 84.2 | 91.4 | 98.6 |
| 97 | 56.8 | 64.1 | 71.4 | 78.8 | 86.1 | 93.5 | 100.8 |
| 98 | 58.3 | 65.8 | 73.2 | 80.6 | 88.1 | 95.5 | 102.9 |
| 99 | 59.9 | 67.4 | 75.0 | 82.5 | 90.0 | 97.6 | 105.1 |
| 100 | 61.5 | 69.1 | 76.8 | 84.4 | 92.0 | 99.7 | 107.3 |
| 101 | 63.1 | 70.8 | 78.5 | 86.3 | 94.0 | 101.7 | 109.5 |
| 102 | 64.7 | 72.5 | 80.3 | 88.2 | 96.0 | 103.8 | 111.7 |
| 103 | 66.3 | 74.2 | 82.2 | 90.1 | 98.0 | 105.9 | 113.9 |
| 104 | 67.9 | 76.0 | 84.0 | 92.0 | 100.0 | 108.1 | 116.1 |
| 105 | 69.6 | 77.7 | 85.8 | 93.9 | 102.1 | 110.2 | 118.3 |
