# Supplementary material for: First-trimester fetal size, accelerated growth in utero, and child neurodevelopment in a cohort study
Source: BMC Med. 2024 Apr 29;22:181. doi: 10.1186/s12916-024-03390-3 (PMC11059611; doi:10.1186/s12916-024-03390-3)
Supplement: Supplementary file 3 — Supplementary Material 3. [file 12916_2024_3390_MOESM3_ESM.docx]

**Table S1. Basic characteristics for the included and excluded mother-child pairs. Values are numbers (percentages) unless stated otherwise.**

| **Characteristics** | **Included, *n* = 2058** | **Excluded, *n* = 3053** | ***P*** |
| --- | --- | --- | --- |
| **Maternal** |  |  |  |
| Mean (SD), age, years | 28.95 (3.50) | 29.04 (3.67) | 0.24 |
| Education levels |  |  | < 0.01 |
| ≤12 years | 372 (18.08) | 642 (21.03) |  |
| >12 years | 1686 (81.92) | 2411 (78.97) |  |
| Annual family income |  |  | < 0.001 |
| <50,000 yuan | 246 (11.95) | 354 (11.60) |  |
| 50,000-100,000 yuan | 750 (36.44) | 934 (30.59) |  |
| >100,000 yuan | 1062 (51.60) | 1765 (57.81) |  |
| Residence area |  |  | < 0.01 |
| Suburban area | 419 (20.36) | 517 (16.93) |  |
| Central urban area | 1639 (79.64) | 2536 (83.07) |  |
| Pre-pregnancy body mass index |  |  | 0.93 |
| Underweight (<18.5 Kg/m2) | 374 (18.17) | 546 (17.88) |  |
| Normal weight (18.5-24.0 Kg/m2) | 1384 (67.25) | 2069 (67.77) |  |
| Overweight or obesity (≥24 Kg/m2) | 300 (14.58) | 438 (14.35) |  |
| Parity |  |  | < 0.001 |
| Nulliparous women | 1705 (82.85) | 2292 (75.07) |  |
| Parous women | 353 (17.15) | 761 (24.93) |  |
| Intake of folic acid supplement |  |  | 0.51 |
| No | 652 (31.68) | 994 (32.56) |  |
| Yes | 1406 (68.32) | 2059 (67.44) |  |
| **Fetal and birth** |  |  |  |
| Mean (SD), gestational age at first-trimester crown to rump length measurement, weeks | 12.52 (0.58) | 12.55 (0.57) | 0.04 |
| Mean (SD), first trimester crown to rump, cm | 6.20 (0.79) | 6.22 (0.76) | 0.28 |
| Sex |  |  | 0.07 |
| Male | 1055 (51.26) | 1643 (53.82) |  |
| Female | 1003 (48.74) | 1410 (46.18) |  |
| Mean (SD), gestation duration, weeks | 39.34 (1.12) | 39.27 (1.22) | 0.06 |
| Mean (SD), birth weight, g | 3332.24 (416.53) | 3337.53 (423.95) | 0.66 |

^a^ Differences in basic characteristics between the included and excluded populations were evaluated using one-way ANOVA tests for continuous variables and chi-square tests for proportions.

**Table S2. Test for non-linear association between exposures and outcomes.**

| Exposure | PDI | | MDI | |
| --- | --- | --- | --- | --- |
|  | Linear^a^ | Spline^b^ | Linear^a^ | Spline^b^ |
| CRL | 0.003 | 0.348 | 0.028 | 0.642 |
| Growth rate | 0.378 | 0.345 | 0.021 | 0.133 |

^a^ *P* value was from generalized additive model using linear fitting.

^b^ *P* value was from generalized additive model using spline fitting.

**Table S3. Fetal size in the second and the third trimester in relation to neurodevelopment scores (*n* = 2058).**

| **Measurements^a^** | **Unadjusted model** | **Adjusted model 1^b^** | **Adjusted model 2^c^** |
| --- | --- | --- | --- |
| **MDI, β (95%CI)** |  |  |  |
| Growth in second-trimester (EFW) | 0.13 (-0.34, 0.60) | 0.81 (0.34, 1.27)* | 0.44 (-0.05, 0.94) |
| Growth in third-trimester (EFW) | 0.59 (0.11, 1.06)* | 1.12 (0.65, 1.59)* | 0.97 (0.50, 1.45)* |
| **PDI, β (95%CI)** |  |  |  |
| Growth in second-trimester (EFW) | 0.83 (0.44, 1.22)* | 0.85 (0.45, 1.24)* | 0.31 (-0.11, 0.73) |
| Growth in third-trimester (EFW) | 0.72 (0.33, 1.12)* | 0.63 (0.23, 1.03)* | 0.41 (-0.00, 0.82) |

Abbreviations: MDI, mental development index; PDI, psychomotor development index; EFW, estimated fetal weight.

Values are regression coefficients (95% confidence interval) estimated by generalized estimating equation models with linear regression based on multiply imputed data. *P*-values were calculated with the Wald t test.

^a^ Fetal size in the second trimester was measured at 23 (±2) weeks, and fetal size in the third trimester was measured at 38 (±2) weeks.

^b^ Adjusted for maternal age, maternal education, annual household income, residence area, pre-pregnancy body mass index, parity, intake of folic acid supplement, and child sex.

^c^ Additionally adjusted for first-trimester CRL Z score based on adjusted model 1.

**P* < 0.05.

**Table S4. Joint analysis of CRL and fetal growth patterns in relation to neurodevelopment scores (*n* = 2058).**

| **Groups^b^** | **MDI, β (95% CI)** | | **PDI, β (95% CI)** | |
| --- | --- | --- | --- | --- |
|  | **Unadjusted** | **Adjusted^a^** | **Unadjusted** | **Adjusted^a^** |
| Restricted first-trimester fetal size |  |  |  |  |
| IFG | Reference | Reference | Reference | Reference |
| IMG | 3.67 (0.95, 6.38)* | 4.10 (1.40, 6.81)* | -0.96 (-3.39, 1.46) | -0.93 (-3.33, 1.46) |
| IAG | 3.67 (0.95, 6.38)* | 6.14 (3.80, 8.49)* | -0.61 (-2.84, 1.61) | -0.85 (-3.12, 1.42) |
| Median first-trimester fetal size |  |  |  |  |
| IFG | Reference | Reference | Reference | Reference |
| IMG | 2.20 (-0.20, 4.61) | 1.80 (-0.53, 4.14) | 0.03 (-1.79, 1.86) | -0.16 (-1.99, 1.67) |
| IAG | 1.23 (-1.09, 3.54) | 1.86 (-0.38, 4.10) | 0.83 (-1.02, 2.69) | 0.23 (-1.65, 2.11) |
| Optimal first-trimester fetal size |  |  |  |  |
| IFG | Reference | Reference | Reference | Reference |
| IMG | -0.43 (-2.87, 2.01) | -0.33 (-2.81, 2.16) | 0.64 (-1.29, 2.56) | 0.70 (-1.24, 2.64) |
| IAG | 0.74 (-1.57, 3.05) | 1.13 (-1.17, 3.42) | 3.05 (0.86, 5.24)* | 3.18 (0.97, 5.38)* |

Abbreviations: MDI, mental development index; PDI, psychomotor development index; IFG, intrauterine faltering growth; IMG, intrauterine median growth; IAG, intrauterine accelerated growth.

Values are regression coefficients (95% confidence interval) estimated by general linear models based on multiply imputed data. *P*-values were calculated with the Wald t test.

^a^ Adjusted for maternal age, maternal education, annual household income, residence area, pre-pregnancy body mass index, parity, intake of folic acid supplement, and child sex.

^b^ Restricted first-trimester fetal size: the lowest tertile of CRL Z score (< -0.38); Median first-trimester fetal size: the middle tertile of CRL Z score (-0.38 to 0.36); Optimal first-trimester fetal size: the highest tertile of CRL Z score (> 0.36); IFG: the lowest tertile of fetal growth rate (< -0.022 EFW Z score/week); IMG: the middle tertile of fetal growth rate (-0.022 to 0.023 EFW Z score/week); IAG: the highest tertile of fetal growth rate (> 0.023 EFW Z score/week).

**P* < 0.05.

**Table S5. Results of sensitivity analyses.**

| **Sensitivity analyses** | **Measurements** | **MDI, β (95% CI)** | **PDI, β (95% CI)** |
| --- | --- | --- | --- |
|  |  | **Adjusted^a^** | **Adjusted^a^** |
| **CRL** | | | |
| Analysis1^b^ | Per increment of Z score | 1.31 (0.19, 2.44)* | 1.39 (0.45, 2.34)* |
| Analysis2^c^ |  | 1.01 (-0.10, 2.13) | 1.25 (0.29, 2.21)* |
| Analysis3^d^ |  | 1.32 (0.22, 2.41)* | 1.55 (0.63, 2.47)* |
| Analysis4^e^ |  | 1.43 (0.50, 2.37)* | 1.34 (0.24, 2.44)* |
| Analysis5^f^ |  | 1.35 (0.41, 2.28)* | 0.94 (-0.16, 2.04) |
| Analysis6^g^ |  | 1.08 (-0.05, 2.21) | 1.19 (0.24, 2.15)* |
| Analysis7^h^ |  | 1.12 (0.06, 2.19)* | 1.21 (0.31, 2.12)* |
| Analysis8^i^ |  | 1.23 (0.17, 2.29)* | 1.30 (0.40, 2.19)* |
| **Fetal growth rate** | |  |  |
| Analysis1^b^ | Per increment of EFW Z score/week | 20.99 (10.78, 31.20)* | 7.92 (-0.44, 16.28) |
| Analysis2^c^ |  | 22.03 (11.85, 32.22)* | 9.58 (0.99, 18.17)* |
| Analysis3^d^ |  | 16.63 (6.46, 26.80)* | 4.69 (-4.00, 13.39) |
| Analysis4^e^ |  | 19.10 (9.12, 29.08)* | 5.92 (-2.52, 14.36) |
| Analysis5^f^ |  | 19.44 (9.48, 29.40)* | 5.61 (-2.88, 14.10) |
| Analysis6^g^ |  | 18.50 (8.28, 28.71)* | 8.22 (-0.31, 16.74) |
| Analysis7^h^ |  | 20.38 (10.93, 29.82)* | 5.69 (-2.46, 13.84) |
| Analysis8^i^ |  | 18.89 (9.62, 28.17)* | 4.74 (-3.52, 12.99) |
| **Fetal growth pattern (restricted first-trimester fetal size)^h^** | | | |
| Analysis1^b^ | IFG | Reference | Reference |
|  | IMG | 4.56 (1.62, 7.49)* | -0.93 (-3.30, 1.44) |
|  | IAG | 6.75 (4.28, 9.21)* | -1.41 (-3.68, 0.86) |
| Analysis2^c^ | IFG | Reference | Reference |
|  | IMG | 3.71 (0.88, 6.54)* | -0.84 (-3.42, 1.73) |
|  | IAG | 6.12 (3.58, 8.65)* | -1.20 (-3.52, 1.12) |
| Analysis3^d^ | IFG | Reference | Reference |
|  | IMG | 3.90 (1.19, 6.61)* | -0.90 (-3.36, 1.57) |
|  | IAG | 5.71 (3.22, 8.21)* | -0.57 (-2.98, 1.84) |
| Analysis4^e^ | IFG | Reference | Reference |
|  | IMG | 4.05 (1.29, 6.81)* | -1.16 (-3.68, 1.35) |
|  | IAG | 6.06 (3.63, 8.49)* | -0.79 (-3.10, 1.53) |
| Analysis5^f^ | IFG | Reference | Reference |
|  | IMG | 3.29 (0.39, 6.19)* | -1.18 (-3.81, 1.44) |
|  | IAG | 5.26 (2.83, 7.70)* | -1.32 (-3.79, 1.14) |
| Analysis6^g^ | IFG | Reference | Reference |
|  | IMG | 3.94 (1.19, 6.70)* | -0.69 (-3.26, 1.88) |
|  | IAG | 5.39 (2.96, 7.83)* | -0.26 (-2.70, 2.18) |
| Analysis7^h^ | IFG | Reference | Reference |
|  | IMG | 4.19 (1.42, 6.96)* | -0.86 (-3.31, 1.58) |
|  | IAG | 6.03 (3.69, 8.36)* | -0.87 (-3.15, 1.41) |
| Analysis8^i^ | IFG | Reference | Reference |
|  | IMG | 4.39 (1.65, 7.13)* | -0.54 (-2.96, 1.87) |
|  | IAG | 6.70 (4.34, 9.05)* | -0.83 (-3.13, 1.47) |

Abbreviations: MDI, mental development index; PDI, psychomotor development index; CRL, crown-lump length; IFG, intrauterine faltering growth; IMG, intrauterine median growth; IAG, intrauterine accelerated growth.

Values are regression coefficients (95% confidence interval) estimated by general linear models. The results of fetal growth rate and fetal growth patterns were based on multiply imputed data. *P*-values were calculated with the Wald t test.

^a^ Adjusted for maternal age, maternal education, annual household income, residence area, pre-pregnancy body mass index, parity, intake of folic acid supplement, and child’s sex.

^b^ The analysis excluded pregnant women with gestational diabetes mellitus (*n* = 172) and pregnancy-induced hypertension (*n* = 43), resulting in 1843 participants included in the analysis.

^c^ The analysis excluded children with low birth weight (< 2500g) (*n* = 35), macrosomia (birth weight ≥ 4000g) (*n* =108), and preterm birth (*n* = 54), resulting in 1861 participants included in the analysis.

^d^ The analysis additionally adjusted for birth weight and gestation duration (*n* = 2058).

^e^ The analysis additionally adjusted for body mass index z-scores at age two years (*n* = 1877).

^f^ The analysis was restricted to the participants with a self-reported regular menstrual cycle (*n* = 1885).

^g^ The analysis was restricted to the participants with a self-reported menstrual cycle of 28 plus or minus three days (*n* = 1842)

^h^ The analysis additionally adjusted for menstrual cycle duration (*n* = 2025).

^i^ The analysis was conducted with the application of inverse probability weighting (*n* = 2058).

^j^ Restricted first-trimester fetal size: the lowest tertile of CRL Z score (< -0.38); IFG: the lowest tertile of fetal growth rate (< -0.022 EFW Z score/week); IMG: the middle tertile of fetal growth rate (-0.022 to 0.023 EFW Z score/week); IAG: the highest tertile of fetal growth rate (> 0.023 EFW Z score/week).

**P* < 0.05.

**Table S6. Basic characteristics for included and excluded mother-child pairs after the application of inverse probability of weighting. Values are percentages unless stated otherwise.**

| **Characteristics** | **Included, *n* = 2058** | **Excluded, *n* = 3053** | ***P*** |
| --- | --- | --- | --- |
| **Maternal** |  |  |  |
| Mean, age, years | 29.01 | 29.00 | 0.96 |
| Education levels |  |  | 0.91 |
| ≤ 12 years | 19.65 | 19.78 |  |
| > 12 years | 80.35 | 80.22 |  |
| Annual family income |  |  | >0.99 |
| <50,000 yuan | 11.70 | 11.72 |  |
| 50,000-100,000 yuan | 33.16 | 33.07 |  |
| >100,000 yuan | 55.14 | 55.21 |  |
| Residence area |  |  | 0.96 |
| Suburban area | 18.25 | 18.30 |  |
| Central urban area | 81.75 | 81.70 |  |
| Pre-pregnancy body mass index |  |  | >0.99 |
| Underweight (<18.5 Kg/m2) | 18.21 | 18.08 |  |
| Normal weight (18.5-24.0 Kg/m2) | 67.30 | 67.46 |  |
| Overweight or obesity (≥24 Kg/m2) | 14.50 | 14.46 |  |
| Parity |  |  | 0.98 |
| Nulliparous women | 78.25 | 78.22 |  |
| Parous women | 21.75 | 21.78 |  |
| Intake of folic acid supplement |  |  | 0.96 |
| No | 32.35 | 32.29 |  |
| Yes | 67.65 | 67.71 |  |
| **Fetal and birth** |  |  |  |
| Sex |  |  | 0.99 |
| Male | 52.74 | 52.76 |  |
| Female | 47.26 | 47.24 |  |
| Mean, gestation duration, weeks | 39.31 | 39.29 | 0.46 |
| Mean, birth weight, g | 3336.94 | 3335.14 | 0.88 |

^a^ Differences in basic characteristics between the included and excluded populations were evaluated using one-way ANOVA tests for continuous variables and chi-square tests for proportions.

**Table S7. Relationships of maternal characteristics and child sex with child neurodevelopment.**

| **Factors** | **MDI, β (95% CI)** | | **PDI, β (95% CI)** | |
| --- | --- | --- | --- | --- |
|  | **Univariate**  **analysis** | **Multivariate**  **analysis^a^** | **Univariate**  **analysis** | **Multivariate**  **analysis^a^** |
| Age, years | 0.08 (-0.18, 0.35) | 0.17 (-0.13, 0.48) | -0.01 (-0.23, 0.21) | -0.23 (-0.49, 0.02) |
| Education levels |  |  |  |  |
| ≤12 years | Reference | Reference | Reference | Reference |
| >12 years | 6.36 (3.94, 8.79)* | 5.14 (2.67, 7.61)* | 0.55 (-1.47, 2.57) | 0.46 (-1.62, 2.55) |
| Annual family income |  |  |  |  |
| <50,000 yuan | Reference | Reference | Reference | Reference |
| 50,000-100,000 yuan | 2.32 (-0.80, 5.44) | 1.87 (-1.22, 4.96) | 1.05 (-1.54, 3.64) | 1.11 (-1.49, 3.72) |
| >100,000 yuan | 5.34 (2.33, 8.35)* | 4.10 (1.06, 7.13)* | 2.21 (-0.27, 4.71) | 2.27 (-0.29, 4.83) |
| Residence area |  |  |  |  |
| Suburban area | Reference | Reference | Reference | Reference |
| Central urban area | 1.20 (-1.13, 3.54) | 0.96 (-1.33, 3.25) | -1.49 (-3.42, 0.44) | -1.64 (-3.58, 0.30) |
| Pre-pregnancy body mass index |  |  |  |  |
| Underweight (<18.5 Kg/m^2^) | 1.82 (-0.66, 4.31) | 2.60 (0.14, 5.06)* | -1.01 (-3.06, 1.05) | -1.08 (-3.16, 1.00) |
| Normal weight (18.5-24.0 Kg/m^2^) | Reference | Reference | Reference | Reference |
| Overweight or obesity (≥24 Kg/m^2^) | -0.88 (-3.59, 1.84) | -0.77 (-3.43, 1.90) | 1.32 (-0.93, 3.56) | 1.36 (-0.89, 3.62) |
| Parity |  |  |  |  |
| Nulliparous women | Reference | Reference | Reference | Reference |
| Parous women | -2.10 (-4.60, 0.39) | -1.98 (-4.74, 0.79) | 2.64 (0.58, 4.70)* | 3.55 (1.22, 5.89)* |
| Intake of folic acid supplement |  |  |  |  |
| No | Reference | Reference | Reference | Reference |
| Yes | 3.00 (0.98, 5.02)* | 2.88 (0.89, 4.86)* | 0.15 (-1.52, 1.82) | 0.21 (-1.47, 1.89) |
| Gestation duration, week | 1.32 (0.48, 2.16)* | 0.99 (0.14, 1.85)* | 0.80 (0.10, 1.49)* | 0.84 (0.12, 1.55)* |
| Gestational diabetes mellitus |  |  |  |  |
| No | Reference | Reference | Reference | Reference |
| Yes | -1.02 (-4.42, 2.37) | 0.23 (-3.18, 3.63) | -1.08 (-3.88, 1.73) | -7.02 (-12.04, -1.99)* |
| Pregnancy-induced hypertension |  |  |  |  |
| No | Reference | Reference | Reference | Reference |
| Yes | -2.83 (-8.82, 3.16) | -0.74 (-6.70, 5.22) | -7.91 (-12.85, -2.96)* | -0.90 (-3.77, 1.97) |
| Child sex |  |  |  |  |
| Male | -7.31 (-9.16, -5.45)* | -7.22 (-9.06, -5.38)* | -1.25 (-2.80, 0.31) | -1.28 (-2.84, 0.27) |
| Female | Reference | Reference | Reference | Reference |

^a^ Multivariate analysis included maternal age, maternal education, annual household income, residence area, pre-pregnancy body mass index, parity, intake of folic acid supplement, gestation duration, gestational diabetes mellitus, pregnancy-induced hypertension, and child’s sex.

**P* < 0.05.

**Table S8. Stratified analyses for predefined effect modification.**

| **Factors** | **Stratification** | **MDI** | | **PDI** | |
| --- | --- | --- | --- | --- | --- |
|  |  | **β (95% CI)** | **P _for interaction_** | **β (95% CI)** | **P _for interaction_** |
| **CRL** |  |  |  |  |  |
| Gender | Male | 1.55 (-0.04, 3.13) | 0.54 | 1.05 (-0.20, 2.30) | 0.34 |
|  | Female | 0.77 (-0.63, 2.17) |  | 1.79 (0.49, 3.09)* |  |
| Pre-pregnancy BMI | Underweight | 1.00 (-1.44, 3.45) | 0.55 | 0.45 (-1.58, 2.47) | 0.16 |
|  | Normal | 1.21 (-0.15, 2.57) |  | 1.94 (0.80, 3.08)* |  |
|  | Overweight or obesity | 0.31 (-2.30, 2.91) |  | -0.25 (-2.37, 1.86) |  |
| Maternal age at delivery | < 30 years | 0.66 (-0.70, 2.03) | 0.35 | 1.38 (0.25, 2.52)* | 0.98 |
|  | ≥ 30 years | 1.69 (-0.08, 3.46) |  | 1.30 (-0.17, 2.77) |  |
| **Growth rate** |  |  |  |  |  |
| Gender | Male | 28.81 (2.82, 54.79)* | 0.47 | 3.53 (-17.02, 24.07) | 0.90 |
|  | Female | 8.51 (-13.63, 30.64) |  | 8.40 (-12.09, 28.89) |  |
| Pre-pregnancy BMI | Underweight | 58.17 (18.19, 98.15)* | 0.07 | 18.11 (-15.43, 51.65) | 0.54 |
|  | Normal | 11.84 (-9.96, 33.64) |  | 4.57 (-13.62, 22.77) |  |
|  | Overweight or obesity | 1.62 (-40.77, 44.01) |  | -8.19 (-42.58, 26.21) |  |
| Maternal age at delivery | < 30 years | 17.41 (-4.26, 39.09) | 0.79 | 9.15 (-8.95, 27.25) | 0.55 |
|  | ≥ 30 years | 18.82 (-10.44, 48.07) |  | -2.22 (-26.58, 22.15) |  |
